# Microbial Evaluation of Fresh, Minimally-processed Vegetables and Bagged Sprouts from Chain Supermarkets

**Published:** 2014-09

**Authors:** Maryam Zare Jeddi, Masud Yunesian, Mohamad Es'haghi Gorji, Negin Noori, Mohammad Reza Pourmand, Gholam Reza Jahed Khaniki

**Affiliations:** ^1^Department of Environmental Health Engineering, School of Public Health, Tehran University of Medical Sciences, Poorsina Street, PO Box 14155-6446, Tehran, Iran; ^2^Center for Air Pollution Research, Institute for Environmental Research, Tehran University of Medical Sciences, Kargar Street, Gol Building, Tehran, Iran; ^3^Department of Food Hygiene, Faculty of Veterinary Medicine, University of Tehran, Azadi Street, Tehran, Iran; ^4^Department of Pathobiology, School of Public Health, Tehran University of Medical Sciences, Poorsina Street, Tehran, Iran

**Keywords:** Foodborne pathogen, Fungal contamination, Microbial safety, Mold, Sprouts, Iran

## Abstract

The aim of this study was to evaluate the bacterial and fungal quality of minimally-processed vegetables (MPV) and sprouts. A total of 116 samples of fresh-cut vegetables, ready-to-eat salads, and mung bean and wheat sprouts were randomly collected and analyzed. The load of aerobic mesophilic bacteria was minimum and maximum in the fresh-cut vegetables and fresh mung bean sprouts respectively, corresponding to populations of 5.3 and 8.5 log CFU/g. *E. coli* O157:H7 was found to be absent in all samples; however,  other *E. coli* strains were detected in 21 samples (18.1%), and *Salmonella* spp. were found in one mung bean (3.1%) and one ready-to-eat salad sample (5%). Yeasts were the predominant organisms and were found in 100% of the samples. *Geotrichum*, *Fusarium,* and *Penicillium* spp. were the most prevalent molds in mung sprouts while *Cladosporium* and *Penicillium* spp. were most frequently found in ready-to-eat salad samples. According to results from the present study, effective control measures should be implemented to minimize the microbiological contamination of fresh produce sold in Tehran, Iran.

## INTRODUCTION

In many parts of the world, including Iran, there is an increasing rate of consumption of raw fresh produce, like vegetables, fruits, and sprouts. This is especially the case for minimally-processed fruits and vegetables, mainly because of changes in the human lifestyle and their tendency towards convenience and spending less time on preparing food (1-3). However, despite their nutritional and healthy characteristics, outbreaks of human infections associated with the consumption of fresh or minimally-processed fruits and vegetables have increased in the recent years due primarily to transmitting various pathogens to humans (4,5). Contamination of these products by pathogenic microorganisms, specifically in leafy green vegetables, poses serious health threats to consumers (6). Freshly-consumed produce can be contaminated with pathogens via being exposed to contamination sources from production on the farm to the point of sale in the market (7). These reported contamination sources are: soil (e.g. manure, faeces, soil microorganisms), dust, water, and handling during pre- or postharvest stages (8). The major sources of postharvest contamination are containers used for transporting the produce, human handling, processing, and storage (9). Avoidance of decontaminating measures before consumption also predisposes fresh produce to remain contaminated with potential human pathogens (10). Moreover, minimally-processed fruits and vegetables are more susceptible to contamination because cutting and slicing damage the natural protective barriers of the intact produce and release nutrients and facilitate growth of microorganisms (1,11,12).

Outbreaks of foodborne diseases linked to the consumption of fresh produce have shown a remarkable increase in the last two decades (13). The Center for Science, in the public interest, recently released a report based on the data from the Centers for Disease Control and Prevention (CDC) and other sources, indicating that green leafy vegetables were associated with 363 outbreaks, including 13,569 announced cases of illness (14). An outbreak of *E. coli* O157:H7 occurred in 26 US states in September 2006, which led to about 200 cases of illness, including some with haemolytic-uraemic syndrome (HUS) and resulted in three deaths (15). Data demonstrated that fresh spinach grown in three Californian counties was responsible for contamination.

*Salmonella* is the leading cause of foodborne diseases throughout the world. In the last few years, outbreaks of S*almonella* have been linked increasingly to consumption of fresh vegetables. This pathogen is the main challenge for the microbiological safety of MPV (16).

Microbiological surveys of MPV products have investigated the occurrence of *Salmonella*, *Escherichia coli*, coliforms, total aerobic and spoilage bacteria, fungi, and yeasts (1,17-22). Most reported counts for total aerobic bacteria ranged between 4 and 8 log CFU/g and between 0.7 and 6 log CFU/g for coliforms. *E. coli* strains have been often observed at low prevalence and low counts. Pathogens, like *E. coli* O157:H7, *Salmonella*, and *L. monocytogenes,* have rarely been found.

To the best of our knowledge, there is no published data on the microbiological quality of this fresh produce in Iran. Therefore, this study represents the first survey in microbial contamination in MPV and sprouts. The aim of this study was to investigate the microbiological quality (both bacterial and fungal contamination) in MPV and sprouts, which are commercially available in Iran, aiming at the future improvement of food safety measures.

## MATERIALS AND METHODS

### Collection of samples

A total of 116 samples of MPV and bagged sprouts were randomly collected from four chain supermarkets during May and July 2012 in Tehran city, Iran. Vegetables and sprouts comprised 20 samples of ready-to-eat salads (containing three to five ingredients, such as lettuce, coleslaw, cucumber, carrots, and tomato), 64 sprout samples (mung and wheat), and 32 samples of fresh-cut vegetables (including seven types of vegetables: Leek, fennel, watercress, basil, and radish). All of the samples were obtained in the original packages before their best-before date, within the shelf-life of up to seven days, as mentioned on the labels. All samples were placed in secure sterile re-sealable plastic bags and transported promptly to the laboratory in ice boxes. Samples that had been unpackaged or visibly damaged were discarded before analysis.

### Bacteriological analysis

Manufacturer's name, type of vegetables, batch number, expiry date, and the type of packaging (modified or non-modified atmosphere) were recorded. The surfaces of the packaging were sterilized before samples were taken out of them. This was done using ethanol-sterile gauze to prevent cross-contamination.

Ten grammes of each sample was weighed and placed in a stomacher bag, and it was diluted in 90 mL of buffered peptone water (BPW) and homogenized for 2 min at 260 rev per minute, using a stomacher (Model 400 circulator, Seward, Norfolk, England). Eight decimal dilutions of the suspension were made in BPW and analyzed for aerobic mesophilic count, *E. coli*, coliforms, molds, and yeasts.

For determination of aerobic mesophilic bacteria, 1 mL of each decimal dilution was added to 12-15 mL plate count agar (PCA), the plates were incubated for 24-48 h in 30 °C, and then the bacterial colonies were counted (23).

To detect *Salmonella* spp., 25 g of each sample was diluted in 225 mL of BPW and homogenized as previously described. Then, these were held for 16-24 h at 35 °C; 0.1 mL of this sample was added to two broth cultures (Selenite cysteine and Tetrathionate broth) that contained iodine and novobiocin, then was incubated at 35 °C and 41.5 °C for 24 h respectively. For isolation and identification, streaking cultures from two mentioned cultures were done by three differential cultures, including brilliant green bile broth (BGBB), xylose lysine deoxycholate agar (XLD agar) and *Salmonella-Shigella* agar (SSA) and incubated for 24-48 h at 37 °C; 1-2 colony(ies) were picked up from each positive plate and cultured on three media (Lysine decarboxylase agar triple, sugar iron agar, and urea broth) to be confirmed (24).

For determining the coliforms, 1 mL of each decimal dilution and 15 mL solid culture medium of violet red bile agar (VRBA) were poured to petridish; after stirring, these were held to fix. Then a thin layer of media were poured to make micro-aerophilic condition, and plates were incubated for 24 h at 35 °C; in the end, red colonies were counted (25). Also, presumptive *E. coli* was determined. At first, 1 mL of primary dilution was added to lauryl sulphate media and incubated at 37 °C for 24 h; if the gas was observed, loopfuls of suspension were transferred to EC broth and kept at 44 °C for 48 h to examine for gas production; gas-positive samples were transferred to PBW to keep at 44 °C for 48 h; indol production was confirmed by using indol reagent, which resulted in red colour that verified presence of *E. coli*. Differentiation of *E. coli* was carried out with IMViC test. *E. coli* strains isolated were plated in Tergitol BCIG agar and Sorbitol-MacConkey agar and incubated at 44±1 °C for the detection of β-glucuronidase and sorbitol-positive strains respectively (26).

For determination of yeasts and molds, 1 mL of each decimal dilution was placed on plate surface that contained Sabouraud dextrose agar (SDA) and distributed by a sterilized swab. Plates were incubated for 5 days at 25 °C. Colonies were counted and expressed as CFU/g; molds were purified on SDA for further subculture for microscopic examination and identification (27).

### Statistical analysis

All of the samples were tested in triplicate. Statistical analyses were estimated using SPSS (version 11.5). In order to analyze the data, standard deviation and mean were calculated. Independent *t*-test were used for determining any statistically significant difference (p<0.05) among two brands of each commodity.

## RESULTS

### Aerobic mesophilic count

Table 1 presents the contamination of fresh-cut vegetables, ready-to-eat salads, and wheat and mung sprouts to aerobic mesophilic bacteria; the average loads were 6.4±0.7, 6.7±0.5, 6.9±0.6, and 7±0.6 log CFU/g respectively.

### Coliforms, E. coli, and Salmonella spp.

The number of total coliforms and pathogens in the vegetables and sprouts are presented in Table 2 and 3. As presented in Table 2, total coliforms were not detected in 13% of the fresh-cut vegetable samples while the rest of the samples were positive. Mung bean sprout samples contained the highest values of thermotolerant coliforms with a mean of 62.5%.

**Table 1.** Aerobic mesophilic counts in examined samples of fresh, minimally-processed vegetables and bagged sprouts

Percentage of samples in the indicated interval							
Mean^a^*	Range^b^	>107	106-107	105-106	<105	No. of samples	Food item
7.0±0.6	6.4-8.5	43.8	56.2	0.0	0.0	32	Mung sprouts
6.9±0.6	5.5-8.4	43.7	50.0	6.3	0.0	32	Wheat sprouts
6.7±0.5	5.5-7.4	25.0	70.0	5.0	0.0	20	Ready-to-eat salads
6.4±0.7	5.3-7.5	18.8	53.1	28.1	0.0	32	Fresh-cut vegetables

*Results are expressed as mean±SD of three repetitions;

^a^Counts are given in terms of log CFU/g of products;

^b^Range in log CFU/g of products

**Table 2.** Total coliform counts in fresh, minimally-processed vegetables and bagged sprout samples obtained from chain supermarkets

Percentage of samples in the indicated interval								
Mean^a^	Range^b^	>105	104-105	103-104	102-103	<102	No. of samples	Food item
4.0±0.9	ND^c^-5.5	15.6	28.1	31.3	9.4	15.6	32	Fresh-cut vegetables
4.7±1.0	2.8-6.5	34.4	40.6	15.6	9.4	0.0	32	Wheat sprouts
4.5±1.1	2.7-6.4	34.4	31.2	31.2	3.2	0.0	32	Mung sprouts
4.0±1.3	1.9-6.0	30.0	20.0	25.0	20.0	5.0	20	Ready-to-eat salads

^a^Results are expressed as mean±SD of three repetitions and the counts are given in terms of log CFU/g of product;

^b^Range in log CFU/g of products;

^c^Not detected

*E. coli* was detected in 3 out of 32 fresh-cut vegetable samples (9.4%), in 6 out of 20 ready-to-eat salad samples (30%), and in 12 of 64 sprout samples (18.7%) (Table 3).

*Salmonella* spp. were not detected in fresh-cut vegetables and wheat sprouts but it was isolated from 1 out of 20 ready-to-eat salad samples (5%) and 1 out of 32 mung bean sprout samples (3.1%) (Table 3).

### Yeasts and molds

The results of mold and yeast counts are presented in Table 4. Yeasts were the predominant organisms and found in 100% of the samples. Molds and yeasts in fresh-cut vegetables, ready-to-eat salads, and sprout samples were in the range of 5.4-7.6, 6.2-7.5, and 6.0-8.5 log CFU/g respectively. Yeast populations were more than 10^6^ CFU/g in the entire ready-to-eat salads and sprout samples. In comparison, fresh-cut vegetables generally contained less amounts of yeasts, with 78% of the samples having counts of <10^6^ CFU/g (Table 4).

Majority of the samples showed molds contamination levels between 10^2^ and 10^4^ CFU/g. The most frequent molds observed in sprout samples comprised *Fusarium, Penicillium,* and *Geotrichum* spp., which were found in 25%, 15.6%, and 10.9% of the samples respectively (Table 5). Among the commodities, wheat sprouts (*Geotrichum* and *Fusarium* spp.), mung sprouts (*Geotrichum* and *Penicillium* spp.), and ready-to eat vegetables (*Cladosporium* and *Penicillium* spp.) constituted the most prevalent molds, and ready-to-eat salads generally contained greater values of molds than fresh-cut vegetable samples. *Cladosporium*, *Penicillium*, *Alternaria,* and *Geotrichum* spp. were the most common filamentous fungi in ready-to-eat salad samples and were present in 35%, 20%, 15%, and 15% respectively.

**Table 3.** Percentage of bacterial groups present in fresh, minimally-processed vegetables and bagged sprout samples from chain supermarkets

*Salmonella* spp.	*E. coli*	Thermotolerant coliforms	Total coliforms	No. of samples	Food item
ND^a^	9.4	34.4	87.5	32	Fresh-cut vegetables
ND^a^	21.9	53.1	100.0	32	Wheat sprouts
3.1	15.6	62.5	100.0	32	Mung sprouts
5.0	30.0	45.0	100.0	20	Ready-to-eat salads

^a^Not detected

**Table 4.** Mold and yeast counts in fresh, minimally-processed vegetables and bagged sprout samples from chain supermarkets

Percentage of samples in the indicated interval								
Mean^a^	Range^b^	>108	107-108	106-107	105-106	<105	No. of samples	Food item
6.9±0.6	6.3-8.5	9.4	31.2	59.4	0.0	0.0	32	Mung bean sprout
6.8±0.6	6.0-8.4	6.2	37.5	56.2	0.0	0.0	32	Wheat sprouts
6.7±0.4	6.2-7.5	0.0	40.0	60.0	0.0	0.0	20	Ready-to-eat salads
6.4±0.6	5.4-7.6	0.0	18.7	59.3	22	0.0	32	Fresh-cut vegetables

^a^Results are expressed as mean±SD of three repetitions and the counts are given in terms of log_10_ CFU/g of product;

^b^Range in log CFU/g of product

**Table 5.** Frequency of fungi isolation in fresh, minimally-processed vegetables and bagged sprout samples from chain supermarkets

Organism	Range (log CFU/g)	Frequency (% contamination samples)
Fresh-cut vegetables (32 samples)		
* Cladosporium*	<100-4.7×103	21.9
* Penicillium*	1.4×10^2^-1.6×103	15.7
* Alternaria*	<100-3.5×103	12.5
* Geotrichum*	<100-2.8×102	6.2
* Trityracium*	2×10^2^-2.5×102	3.1
* Aspergillus*	<100-3×102	6.2
Wheat sprouts (32 samples)		
* Cladosporium*	<100-4×103	15.6
* Penicillium*	<100-3.7×103	9.4
* Alternaria*	<100-1.9×103	9.4
* Geotrichum*	<100-4.5×104	21.9
* Rhizopus*	<100-2.4×102	6.2
* Fusarium*	<100-9×103	12.5
Mung bean sprouts		
* Cladosporium*	<100-4.2×103	12.5
* Penicillium*	<100-1.7×105	12.5
* Alternaria*	100-1.8×102	3.1
* Geotrichum*	<100-3.8×105	28
* Rhizopus*	<100-2.6×103	9.4
* Fusarium*	<100-5.2×103	18.7
Ready-to-eat salads (20 samples)		
* Cladosporium*	<100-1.3×104	35
* Penicillium*	<100-2.6×104	20
* Alternaria*	<100-4.4×103	15
* Geotrichum*	<100-3.3×103	15
* Trityracium*	<100-7×102	10
* Aspergillus*	100-2.4×103	5

## DISCUSSION

Microbial quality of fresh, minimally-processed vegetables and bagged sprouts obtained from chain supermarkets in Tehran city of Iran was determined. In this study, significant differences were not observed among commercial brands. With respect to the national standards of Iran (28), only 3% of the sprout samples (mung sprout and wheat germ), 28.1% of the fresh-cut vegetable samples, and 5% of the ready-to-eat salad samples, which contained less than 6.0 log CFU/g aerobic mesophilic bacteria, can be considered safe for consumption. The average count of aerobic mesophilic bacteria observed in this study was similar to that observed by Seow *et al*. (29) who reported that the mean count of aerobic mesophilic (6.5 log CFU/g) ranged from 5.8 to 7.3 log CFU/g, in 13 packs of salad. Similarly, Valentin-Bon *et al*. (20) conducted a study under the supervision of Food and Drug Administration (FDA) in the United States (USA) on 100 bagged lettuce and spinach mixes and revealed that the average count of aerobic mesophilic bacteria was 7 log CFU/g, ranging from <4 to 8.3 log CFU/g.

Abadias *et al*. (1) reported that the average count of aerobic mesophilic bacteria in 236 samples of fresh-cut vegetables was in the range of 4.3 to 8.9 log CFU/g. In another study, the microbial quality of fresh-cut vegetables and fruits was evaluated by Gómez-Govea *et al*. (2). The results indicated that the levels of mesophilic organisms ranged from 10 to 10^7^ CFU/g.

As mentioned on the labels of sprouts and fresh-cut vegetables, it is recommended to wash these products before consumption. Our analysis showed that the maximum reduction of 0.5 log CFU/g in the microbial load is achieved after this washing step, which was not remarkable (data not shown). Because salads are directly used, appropriate and effective washing could not play a substantial role in decreasing the microbial load and caused a maximum of 2 log CFU/g reduction in the fresh-cut vegetables (30).

Due to the favorauble conditions present in sprout samples, including high humidity, high temperatures, and suitable pH and nutrients, microbial population can proliferate fast, especially during sprouting (29,31). Martínez-Villaluenga *et al*. (31) evaluated the role of germination in the microbial loads of broccoli seeds and reported that the amount of aerobic mesophilic bacteria, total and thermotolerant coliforms increased approximately by 2 and 3 log CFU/g after five days of germination respectively. Hence, these products are exposed to higher pathogenic contamination, such as *E. coli* and *Salmonella* spp. compared to vegetables. Therefore, preventive technical approaches should be implemented to ensure safety of sprouts (32).

de Oliveira *et al*. (21) assessed the microbial quality of 162 MPV samples and detected *E. coli* in 53.1% of the samples, *Salmonella* spp. in 1.2% of the samples, and total and thermotolerant coliforms in 81.5% and 66% of the samples respectively (21). Seow *et al*. (29) mentioned that the average load of coliforms in 13 ready-to-eat salad samples and 14 bean sprout samples were 5.2 and 5.7 log CFU/g respectively, and all samples contained populations greater than 4 log CFU/g. Aycicek *et al*. (7) pointed out that there are some factors influencing high load of coliform in leafy vegetables, including large surfaces exposed to contamination, intense use of untreated manure during preharvest, and more handling steps during postharvest. In the present study, the population of total coliforms in 95% of the samples exceeded 2 log CFU/g (national standard). In spite of the absence of *E. coli* O157:H7 in the samples of this study, *E. coli* strains were detected in 9.4% of fresh-cut vegetable samples, in 30% of ready-to-eat salad samples, and in 18.7% of sprout samples. Also, *E. coli* O157:H7 has not been isolated in several studies (1,2,18,22,29). The prevalence of *E. coli* found in the present study was lower than that in the study by Prado *et al.* (33), that reported *E. coli* contamination in 30% of the MPV samples. Based on a report by Abadias *et al*. (1), *E. coli* contamination was found in 7.1% of the whole vegetable samples and 11.4% of the fresh-cut vegetable samples; only two fresh-cut vegetable samples (0.8%) had *E. coli* counts exceeding 100 MPN/g. In another study (34) on faecal indicator in fresh produce, *E. coli* strains were identified in 8.1% of carrots, lettuce, green onions, and spinach samples.

In several similar studies on the microbial quality of vegetables, *E. coli* and *Salmonella* spp. were either not detected in any of the samples, or these were detected in only one sample (2,22,29,35). *Salmonella* contamination in this study is low when compared with the results of the study by Bruno *et al*. (36) who evaluated the microbial quality of samples of MPV commercialized in northeast Brazil (46.7%). However, our results are consistent with those of the study by Fröder *et al*. (17) who worked on minimally-processed leafy vegetables commercialized in São Paulo, Brazil (3%). In a study conducted by Viswanathan and Kaur (37) on 120 samples of fresh vegetables, fresh-cut fruits, and sprouts, faecal *E. coli* strains were verified in 31.9% of vegetables and 66.6% of sprout samples whereas *Salmonella* spp. were isolated from one sprout sample (brown mung). In our study, in addition to *E. coli* and *Salmonella*, several Gram-negative bacteria, such as *Enterobacter*, *Klebsiella*, and *Erwinia,* were found in the ready-to-eat vegetable packages, which had presumptive colonies on chromogenic agars; this is consistent with the results from the study of Abadias *et al*. (1) and Seow *et al*. (29). *Enterobacter*, *Erwinia* spp. and other non-faecal coliforms bacteria have long been recognized as common organisms in fresh produce, such as lettuce and fresh-cut salads (38). However, their presence is not considered a public health threat but may result in spoilage of vegetables and fruits, such as *Erwinia* spp., which produce pectolytic enzymes and soften vegetable tissues. Therefore, level of faecal organisms, such as *E. coli,* is a better indicator of quality of fresh produce, and this could explain why this organism has been included as a hygienic criterion in the new EU regulation (1).

In contrast to the present study, Badosa *et al*. (39) reported yeast and mold counts in most of the vegetable samples, ranging from 4 to 7 log CFU/g. Acevedo *et al*. (40) also detected molds in the levels of 4.5×10^4^ CFU/g in salad samples. They reported the frequent presence of *Penicillium*, *Aspergillus,* and *Fusarium* spp. in salads. Although organisms, such as *Rhizopus stolonifer* and *Paecilomyces,* were found at low levels in sprout samples, these can grow fast and cause spoilage in short time. In addition to the differences in local sanitary conditions from farm to the market, various methodologies used in these studies could yield different results.

Many of the isolated molds belonged to toxigenic genera, such as *Penicillium*, *Alternaria*, and *Fusarium* spp. The high numbers of *Penicillium* spp. in bean sprout samples and their ability to grow at refrigeration temperatures indicate a potential for mycotoxin production of these foodstuff during marketing (19). According to some authors, the high density of mycotoxin-producing molds generally correspond to poor cleaning practices and/or use of unhygienic techniques and contaminated equipment (16,41).

Cantwell and Kasmire (42) referred a significant increase in the number of bacteria during the cutting process, automatic filling, and packaging lines of lettuce. It seems that a clean product can be re-infected after passing through stages in which there is the possibility of remaining vegetables, such as cutting, filling in equipment, and packaging. Several studies conducted on the production of these items in the workshops (43-45) showed that the surfaces of cutting tools, peeling machines, centrifuge systems, and the sorting room-air are the most polluted parts and that secondary contamination of products can occur anywhere in the production and contribution line.

None of the heat or freezing process is used in the production of fresh ready-to-eat vegetables for removing microorganisms, and the common processes for preparing vegetables with minimal process include sorting, cleaning, disinfecting, peeling, cutting, drying, packing, cold storage, and transport to distribution centres, depending on the products (18,46). In most cases, failure of the cold chain management reduces the shelf-life of the products (46).

However, disinfection with chlorine is the main method for reducing pathogenic organisms (13) but several factors, including bioﬁlm formation by bacteria, internalization of pathogens within plant tissue, and the hydrophobicity of plant surfaces, decrease its effectiveness (47). There is a need to find alternative methods for preserving fresh-cut vegetables in order to improve the efficacy of washing treatments. Alternatives or modified methods have been proposed, including irradiation (48,49), ozone (50,51), bacteriophages, antagonistic bacteria (52,53), and essential oils (54,55). However, none has yet gained widespread acceptance by the industry. For this reason, there is a need to develop alternative methods and, subsequently, the need to develop markers to measure the efficacy of these alternatives.

### Conclusions

Although the number of samples studied was small for some items due to sampling limitations, we believe this project provides a general overview of the microbiological quality of MPV and sprouts commercialized in Tehran, Iran. Due to high values of contamination found in MPV and sprout samples, it cannot be concluded that the fresh products analyzed have appropriate hygienic quality. MPV are subjected to various conditions during growth, harvest, preparation, packaging, and distribution that could cause increased contamination. These products are only treated by disinfection, which does not assure complete removal of microorganisms. Hence, these results suggest that measures, including good agricultural practices (GAP), good manufacturing practices (GMP), and Hazard Analysis and Critical Control Points (HACCP), should be implemented to reduce the risk of microbial contamination from farm-to-fork and to assure safe products.

## ACKNOWLEDGEMENTS

This research was financially supported by Tehran University of Medical Sciences & Health Services, with Grant No. 16811.

## References

[1] Abadias M, Usall J, Anguera M, Solsona C, Viñas I (2008). Microbiological quality of fresh, minimally-processed fruit and vegetables, and sprouts from retail establishments. Int J Food Microbiol.

[2] Gómez-Govea M, Solís-Soto L, Heredia N, García S, Moreno G, Tovar O (2012). Analysis of microbial contamination levels of fruits and vegetables at retail in Monterrey, Mexico. J Food Agric Env.

[3] Ragaert P, Devlieghere F, Debevere J (2007). Role of microbiological and physiological spoilage mechanisms during storage of minimally processed vegetables. Postharvest Biol Technol.

[4] Beuchat LR (2002). Ecological factors influencing survival and growth of human pathogens on raw fruits and vegetables. Microbes Infect.

[5] Altekruse SF, Swerdlow DL (1996). The changing epidemiology of foodborne diseases. Am J Med Sci.

[6] WHO/FAO (2008). Microbiological hazards in fresh leafy vegetables and herbs: meeting report.

[7] Aycicek H, Oguz U, Karci K (2006). Determination of total aerobic and indicator bacteria on some raw eaten vegetables from wholesalers in Ankara, Turkey. Int J Hyg Environ Health.

[8] Eni AO, Oluwawemitan IA, Oranusi S (2010). Microbial quality of fruits and vegetables sold in Sango Ota, Nigeria. Afr J Food Sci.

[9] Rediers H, Claes M, Peeters L, Willems KA (2009). Evaluation of the cold chain of fresh-cut endive from farmer to plate. Postharvest Biol Technol.

[10] Burnett SL, Beuchat LR (2001). Human pathogens associated with raw produce and unpasteurized juices, and difficulties in decontamination. J Ind Microbiol Biotechnol.

[11] Harris LJ, Farber JN, Beuchat LR, Parish ME, Suslow TV, Garrett EH (2003). Outbreaks associated with fresh produce: incidence, growth and survival of pathogens in fresh and fresh-cut produce. Comp Rev Food Sci Food Safety.

[12] de Oliveira Silva E, do Socorro Rocha Bastos M, Wurlitz NJ, de Jesus Barros Z, Mangan F, Minimal processing Rodrigues S, Fernandes FAN (2012). Advances in fruit processing technologies.

[13] Warriner K, Huber A, Namvar A, Fan W, Dunfield K (2009). Recent advances in the microbial safety of fresh fruits and vegetables. Adv Food Nutr Res.

[14] Klein S, Witmer J, Tian A, DeWaal CS (2009). The ten riskiest foods regulated by the U.S. Food and Drug Administration.

[15] U.S. Food and Drug Administration Spinach and E. coli outbreak. Silver Spring, MD: U.S. Food and Drug Administration, 2006. (http://www.fda.gov/newsevents/publichealthfocus/ucm179124.htm, accessed on 17 February 2013).

[16] Sant'Ana AS, Landgraf M, Destro MT, Franco BDGM (2011). Prevalence and counts of Salmonella spp. in minimally processed vegetables in São Paulo, Brazil. Food Microbiol.

[17] Fröder H, Martins CG, De Souza KLO, Landgraf M, Franco BDGM, Destro MT (2007). Minimally processed vegetable salads: microbial quality evaluation. J Food Prot.

[18] Sagoo SK, Little CL, Ward L, Gillespie IA, Mitchell RT (2003). Microbiological study of ready-to-eat salad vegetables from retail establishments uncovers a national outbreak of salmonellosis. J Food Prot.

[19] Tournas VH (2005). Moulds and yeasts in fresh and minimally processed vegetables, and sprouts. Int J Food Microbiol.

[20] Valentin-Bon I, Jacobson A, Monday SR, Feng PCH (2008). Microbiological quality of bagged cut spinach and lettuce mixes. Appl Environ Microbiol.

[21] de Oliveira MA, de Souza VM, Bergamini AMM, de Martinis ECP (2011). Microbiological quality of ready-to-eat minimally processed vegetables consumed in Brazil. Food Control.

[22] Johannessen GS, Loncarevic S, Kruse H (2002). Bacteriological analysis of fresh produce in Norway. Int J Food Microbiol.

[23] International Organization for Standardization (2013). Microbiology of food and animal feeding stuffs—hori-zontal method for the enumeration of microorganisms. Part 1: colony count at 30 degrees C by the pour plate technique. Geneva: International Organization for Standardization.

[24] International Organization for Standardization (2002). Microbiology of food and animal feeding stuffs—horizontal method for the detection of Salmonella spp..

[25] International Organization for Standardization (2005). Microbiology of food and animal feeding stuffs—detection and enumeration of presumptive Escherichia coli: most probable number technique.

[26] International Organization for Standardization. (2006). Microbiology of food and animal feeding stuffs—horizontal method for the detection and enumeration of coliforms—most probable number technique.

[27] International Organization for Standardization. (2008). Microbiology of food and animal feeding stuffs—horizontal method for the enumeration of yeasts and moulds. Part 1:colony count technique in products with water activity greater than 0.95.

[28] Institute of Standards and Industrial Research of Iran. (2007). Packed fresh vegetable to ready for use: specifications and test methods.

[29] Seow J, Ágoston R, Phua L, Yuk H-G (2011). Microbiological quality of fresh vegetables and fruits sold in Singapore. Food Control.

[30] Seymour IJ (1999). Review of current industry practice on fruit and vegetable decontamination.

[31] Martínez-Villaluenga C, Frías J, Gulewicz P, Gulewicz K, Vidal-Valverde C (2008). Food safety evaluation of broccoli and radish sprouts. Food Chem Toxicol.

[32] Taormina PJ, Beuchat LR, Slutsker L (1999). Infections associated with eating seed sprouts: an international concern. Emerg Infect Dis.

[33] Prado SPT, Ribeiro EGA, Capuano DM, de Aquino AL, Rock GM, Bergamini AMM (2008). Microbiological and parasitic quality and labeling adequacy of minimally-processed vegetables, commercialized in Ribeirão Preto, SP/Brazil. Rev Inst Adolfo Lutz.

[34] Bohaychuk VM, Bradbury RW, Dimock R, Fehr M, Gensler GE, King RK (2009). A microbiological survey of selected Alberta-grown fresh produce from farmers’ markets in Alberta, Canada. J Food Prot.

[35] Johnston LM, Jaykus L-A, Moll D, Martinez MC, Anciso J, Mora B (2005). A field study of the microbiological quality of fresh produce. J Food Prot.

[36] Bruno LM, de Queiroz AAM, Andrade APC, Vasconcelos NM, De Fátima Borges M (2005). [Avaliação microbiológica de hortaliças e frutas minimamente processadas Comercializadas em Fortaleza (CE)]. Bol Centro Pesqui Process Aliment.

[37] Viswanathan P, Kaur R (2001). Prevalence and growth of pathogens on salad vegetables, fruits and sprouts. Int J Hyg Environ Health.

[38] Soriano JM, Rico H, Moltó JC, Mañes J (2000). Assessment of the microbiological quality and wash treatments of lettuce served in University restaurants. Int J Food Microbiol.

[39] Badosa E, Trias R, Parés D, Pla M, Montesinos E (2008). Microbiological quality of fresh fruit and vegetable products in Catalonia (Spain) using normalised plate-counting methods and real time polymerase chain reaction (QPCR). J Sci Food Agric.

[40] Acevedo L, Mendoza C, Oyón R (2001). [Total and fecal coliforms, some enterobacteria staphylococcus sp. and moulds in salads for hot dogs sold in Maracay, Venezuela]. Arch Latinoam Nutr.

[41] Lynch MF, Tauxe RV, Hedberg CW (2009). The growing burden of foodborne outbreaks due to contaminated fresh produce: risks and opportunities. Epidemiol Infect.

[42] Cantwell M, Kasmire R, Kader AA (2002). Postharvest handling systems: flower, leafy, and stem vegetables. Postharvest technology of horticultural crops.

[43] Lehto M, Kuisma R, Määttä J, Kymäläinen H-R, Mäki M (2011). Hygienic level and surface contamination in fresh-cut vegetable production plants. Food Control.

[44] López-Gálvez F, Allende A, Selma MV, Gil MI (2009). Prevention of Escherichia coli cross-contamination by different commercial sanitizers during washing of fresh-cut lettuce. Int J Food Microbiol.

[45] Allende A, Selma MV, López-Gálvez F, Villaescusa R, Gil MI (2008). Impact of wash water quality on sensory and microbial quality, including Escherichia coli cross-contamination, of fresh-cut escarole. J Food Prot.

[46] Barth M, Hankinson TR, Zhuang H, Breidt F, Sperber WH, Doyle MP (2009). Microbiological spoilage of fruits and vegetables. Compendium of the microbiological spoilage of foods and beverages. Food microbiology and food safety.

[47] Whipps JM, Hand P, Pink DAC, Bending GD (2008). Human pathogens and the phyllosphere. Adv Appl Microbiol.

[48] Gomes C, Da Silva P, Chimbombi E, Kim J, Castell-Perez E, Moreira RG (2008). Electron-beam irradiation of fresh broccoli heads (Brassica oleracea L. italica). LWT-Food Sci Technol.

[49] Mahmoud BSM (2010). The effects of X-ray radiation on Escherichia coli O157:H7, Listeria monocytogenes, Salmonella enterica and Shigella flexneri inoculated on whole Roma tomatoes. Food Microbiol.

[50] Habibi Najafi MB, Haddad Khodaparast MH (2009). Efficacy of ozone to reduce microbial populations in date fruits. Food Control.

[51] Selma MV, Beltrán D, Allende A, Chacón-Vera E, Gil MI (2007). Elimination by ozone of Shigella sonnei in shredded lettuce and water. Food Microbiol.

[52] Scolari G, Vescovo M (2004). Microbial antagonism of Lactobacillus casei added to fresh vegetables. Ital J Food Sci.

[53] Trias R, Bañeras L, Badosa E, Montesinos E (2008). Bioprotection of Golden Delicious apples and Iceberg lettuce against foodborne bacterial pathogens by lactic acid bacteria. Int J Food Microbiol.

[54] de Azeredo GA, Stamford TLM, Nunes PC, Gomes Neto NJ, de Oliveira MEG, de Souza EL (2011). Combined application of essential oils from Origanum vulgare L. and Rosmarinus officinalis L. to inhibit bacteria and autochthonous microflora associated with minimally processed vegetables. Food Res Int.

[55] Karagözlü N, Ergönül B, Özcan D (2011). Determination of antimicrobial effect of mint and basil essential oils on survival of E. coli O157:H7 and S. typhimurium in fresh-cut lettuce and purslane. Food Control.

